# Experiences of internationally educated nurses working with older adults: A scoping review protocol

**DOI:** 10.1371/journal.pone.0307795

**Published:** 2024-10-03

**Authors:** Kristina M. Kokorelias, Marianne Saragosa, Reham Abdelhalim, Eleni Philippopoulos, Ann Vo

**Affiliations:** 1 Division of Geriatric Medicine, Department of Medicine, Sinai Health System and University Health Network, Toronto, Ontario, Canada; 2 Department of Occupational Science & Occupational Therapy, Temerty Faculty of Medicine, University of Toronto, Toronto, Ontario, Canada; 3 Rehabilitation Sciences Institute, Temerty Faculty of Medicine, University of Toronto, Toronto, Ontario, Canada; 4 National Institute on Ageing, Toronto Metropolitan University, Toronto, Ontario, Canada; 5 Lunenfeld-Tanenbaum Research Institute, Sinai Health, Toronto, Ontario, Canada; 6 Burlington OHT, Burlington, Ontario, Canada; 7 Sidney Liswood Health Science Library, Sinai Health, Toronto, Ontario, Canada; Bangladesh Open University, BANGLADESH

## Abstract

**Background:**

Canada, like many other jurisdictions worldwide, is facing a nursing shortage. At the same time, high-income countries are facing a rapidly ageing and more complex older adult population. Demands for more responsive health care services are driving systems of care to meet the evolving needs of the ageing population. Internationally-educated nurses (IENs) can help fill gaps in the care of older adults, but may need considerable support to work in new social and health care environments. However, the experiences of IENs within the geriatric care literature have not been comprehensively reviewed. This protocol will outline a scoping review to determine: (1) what is known about the experiences and support needs of IENs in geriatric healthcare settings within high income countries? (2) what are current supportive-pathways for IENs in geriatric practice settings? And (3) what are the research gaps in the existing literature on the experiences and support needs of IENs within the context of geriatrics?

**Methods:**

A scoping review will be conducted guided by the methodological framework set out by Arksey and O’Malley (2005) and later advanced by Levac, Colquhoun and O’Brien (2010). The search strategy will be applied to seven databases (MEDLINE, PubMed (non-MEDLINE records), PsycINFO, PsychArticles, CINAHL, Scopus, Web of Science). Grey literature will be searched using Google search engines, targeted websites and consultation with content experts. Articles of any publication date will be included. A two-stage screening process will be conducted in duplicate (i.e., two reviewers per stage) to determine eligible articles. Data from eligible articles will be extracted using a piloted charting form. Extracted data will be analyzed using thematic and descriptive analyses.

**Discussion:**

The findings of the upcoming will highlight opportunities and recommendations to inform future research and support training to support IENs working with older adults within high income countries. Publication, presentations and stakeholder meetings will disseminate our findings.

## Introduction

Nurses comprise the largest health care worker population within high-income countries [1]. At the same time, health human resource shortages in the context of nursing specialists in countries such as Canada [2, 3], the United States [4], and elsewhere [5] have received significant attention, particularly after the COVID-19 pandemic [6]. Nurses who immigrate, or return, to Canada with their nursing degrees stand at the crossroads of a complex set of factors, including barriers to licensure [7–9] and discrimination, marginalization, devaluing of their credentials, lack of support, and lack of professional development and training support, language and communication challenges and differences in work culture, responsibilities, and expectations [10].

Concurrent with a health human resource shortage, geriatric care is significantly increasing within high income countries [11–14]. Yet, there is an ever-growing gap in the capacity of a knowledgeable and skilled health care workforce to provide effective and appropriate care to older adults with increasing physical, social, mental, and cognitive needs associated with ageing [15–19]. In particular, rural areas report the lowest number of geriatric care providers [16]. The lack of geriatric specialists can be even direr for rural older persons, as rural older adults are more likely to have higher rates of chronic disease and disability [20] and fewer economic resources [21] compared to older adults living in urban settings. The integration of internationally educated nurses (IENs) within existing health care teams caring for older adults, may be one possible solution to meeting the needs of a rapidly ageing and complex population [21, 22]. Within this context, IENs refer to individuals who have completed their nursing education outside the country where they intend to practice [21, 22]. These nurses might undergo assessments or additional training to meet local licensing or certification requirements before practicing in their host country [21, 22].

Caring for the geriatric population is among the most challenging tasks for nurses who report concerns over providing care to elderly patients with geriatric syndromes and multiple morbidities [23–25]. A literature review concluded that nurses often have both positive and negative attitudes toward the care of older adults [1], which can result in ageist perceptions that can affect the well-being of older adults in their care [26–28]. Ageism in the context of nursing is believed to be the result of nurses’ poor knowledge of the unique needs of older adults [29]. Thus, an increase in the training of nurses in the context of geriatric care has been recommended to help improve their confidence and overcome ageist attitudes towards older patients [15, 30–32]. However, existing literature has tended to focus on current national nurses and nursing students, without consideration of the unique training needs of IENs (e.g., [33]).

Existing research has found that IENs would benefit from workplace training and support [34, 35] to help them adapt to a new nursing culture of care [36], but that clinical educators are unable to support IENs (e.g., no education about the strengths and challenges experienced by IENs) [37]. Moreover, existing health care teams are often unaware of religious, cultural, and experiential differences (e.g., interprofessional teams, patient-centered care) that IENs experience when providing care in a new country [37, 38]. Health care organizations also lack the understanding of issues of race and gender discrimination IENs experience that hinder their ability to provide high-quality care [39]. Literature reviews have found there is a need support IENs for adaptations in the cultural context of the work environment [40–44]. None of these reviews have been focused on nurses working with older patients, failing to consider unique experiences and support needs when working with older adult populations [45, 46]. Moreover, IENs are not a homogenous group [47]; thus, their experiences in different geriatric health care teams may differ across settings and contexts [48–50]. While efforts have begun to create a research agenda for integrating IENs based on scoping review methodology, this review was not focused on experiences within the context of geriatrics nor support needs [42]. Moreover, the recommendations contained in this review were based on published literature between 2000–2013 and thus may lack relevance given the rapidly evolving health care landscape (e.g., human resource management policies, accelerated licensing programs [51, 52] and health care reforms that may influence the experiences of IENs [5, 50, 53]. Consequently, much more research and programing are needed before IENs can fully integrate into the geriatric health care workforce [34, 54].

Despite the potential role IENs can have within the context of geriatrics care, current research on this topic still needs to be expanded in scope. To account for this gap in research, a comprehensive overview of what exists and where future research should be focused is needed. Thus, the current scoping review protocol outlines a plan to contribute to the existing literature by highlighting: (1) what is known about the experiences and support needs of IENs in geriatric health care settings within high income countries? (2) what are current supportive interventions for IENs in geriatric practice settings? And (3) what are the research gaps in the existing literature on the experiences and support needs of IENs within the context of geriatrics? In doing so, the proposed review’s findings will help inform future research studies, and evidence-informed training programs, addressing the support needs of IENs within the context of geriatrics. The selection of the high-income country in this study was primarily driven by the wealth of available data and to ensure a comparable healthcare system for robust analysis. Nonetheless, future investigations aim to expand this research to encompass varying economic contexts, acknowledging the potential adaptability and relevance of interventions across diverse socioeconomic landscapes.

## Methods

This paper presents a protocol for a scoping review. A scoping review methodology is most appropriate because of the broad nature of the intent of the study, which is to provide an overview of the studies exploring the experiences, support needs and interventions for IENs within the context of geriatrics [55]. This scoping review’s conduct will be guided by Arksey and O’Malley’s scoping review stages [56] that were later advanced by Levac et al. [57] and have been used in numerous review protocols [58]. The conduct of this review will follow the Preferred Reporting Items for Systematic reviews and Meta-Analyses extension for Scoping Reviews (PRISMA-ScR) guidelines [59] to enhance reporting quality and ensure procedural rigour. We will begin this review in February 2024and anticipate disseminating the results within a year.

### Stage 1: Identifying the research question (RQ)

This review aims to learn from the existing geriatrics literature on the experiences of IENs to inform future research and program considerations. This proposed review will address the following RQs: (1) what is known about the experiences and support needs of IENs in geriatric healthcare settings? (2) what are current supportive interventions for IENs in geriatric practice settings? And (3) what are the research gaps in the existing literature on the experiences and support needs of IENs within the context of geriatrics?

The research questions were developed by the research team and the public was not involved in informing these questions. Thus, the questions were informed by the positionality of the research team regarding the research subject.

### Positionality of the research team

This scoping review informs a larger program of research in which the authors are engaged with examining the experiences, support and training needs of Canadian IENs working with older adult populations with the overarching goal of supporting efforts to build national capacity in the geriatric IEN nursing field. Three authors are PhD-trained in qualitative health service research (KMK, MS, RA). Two of the authors are Canadian-trained nurses (MS and AV). One author is an internationally-trained physician (RA). One author works within clinical geriatric nursing (AV).

### Stage 2: Identifying relevant studies

Peer-reviewed and grey literature will be identified using a search strategy created between an Information Specialist and Health Science librarian (EP), and the review team. To ensure we comprehensively capture relevant literature, we define experiences and support needs broadly and heterogeneously to include informational, emotional, training, instrumental (e.g., training in care activities), and appraisal (e.g., receiving feedback about care provided) needs [60]. Further, no date limitations will be imposed. This decision was made to facilitate a comprehensive exploration of historical and contemporary literature, ensuring an inclusive synthesis of evidence to capture the evolution and breadth of research pertaining to the experience of IENs. However, the searches will be restricted to studies available in the English language due to resource constraints.

The search for peer-reviewed literature will be conducted in in seven databases PubMed, PsycINFO, PsychArticles, CINAHL, Scopus, Web of Science, and EThOS (Electronic Theses Online Services). Grey literature will be searched using Google search engines, targeted websites and consultation with content experts (e.g., nursing educators, nurses, and nurse managers) known to the research team. Targeted websites include OpenGrey, ProQuest Sociological Abstracts and ProQuest ERIC, Healthcare Management Information Consortium, Open grey repository, Proceedings First, Canada Health and Council for Allied Health Professions Research. Grey literature includes government reports and documents and open access theses. We will only review the first hundred pages of each search’s results, as done in previous reviews [16, 61].

A preliminary search strategy has been developed between the Information Specialist, and Health Science librarian (EP) and primary author (KMK) to inform the final search strategy **(See S1 File)**. This preliminary search was trialed in OVID MEDLINE on April 17, 2023. Once a semi-final search strategy is conducted, the search will be peer-reviewed according to the peer review of electronic search strategies [62]. Once the search has been peer-reviewed, the Information Specialist and Health Science librarian will translate the final search strategy to other databases (i.e., PubMed (non-MEDLINE records), PsycINFO, PsychArticles, CINAHL, Scopus, Web of Science), have all members of the research team approve the strategy and run the searches in the databases.

To identify studies that were not captured within the searches, we will hand-search reference lists of included articles and conduct forward screening (i.e., reviewing the citation index that cites eligible studies included in the review) [63].

### Stage 3: Study selection

Articles identified from the search will first be deduplicated using the Bramer method in Endnote reference management software [64, 65]. Next, deduplicated articles will be imported into Covidence software to help manage the review process [66, 67].

Articles will be included if they meet the following criteria: a) the population includes IENs involved in the care of an older adult and at least 50% of the sample includes IENs; b) the nurse is employed within the public healthcare system; c) the topic of interest is on the experiences and needs as perceived by IENs caring for older adults in any setting including community, rehabilitation, long-term, acute and primary care; d) the publication was from a ‘high-income country’ as classified by the World Bank [68]. **See Table 1** for the inclusion criteria in accordance with the Joanna Briggs Institute (JBI) framework for Population, Concept and Context (PCC) [69].

**Table 1:** Inclusion and exclusion criteria.

	Inclusion	Exclusion
Population	> 50% of the sample includes IENs. IENs encompass nurses educated outside the country where they intend to practice, were included as subjects in our study’s criteria.	Undergraduate nurses who have yet to practice.
Concept	Experiences and needs as perceived by IENs caring for older adults in any setting including community, rehabilitation, long-term, acute and primary care	
Context	The nurse is employed within the public healthcare system within a high-income country. High income countries include: Andorra, Greece, Poland, Antigua and Barbuda, Greenland, Portugal, Aruba, Guam, Puerto Rico, Australia, Hong Kong SAR China, Qatar, Austria, Hungary, San Marino, Bahamas, Iceland, Saudi Arabia, Bahrain, Ireland, Seychelles, Barbados, Isle of Man, Singapore, Belgium, Israel, Sint Maarten (Dutch part), Bermuda, Italy, Slovak Republic, British Virgin Islands, Japan, Slovenia, Brunei Darussalam, Korea Rep., Spain, Canada, Kuwait, St. Kitts and Nevis, Cayman Islands, Latvia, St. Martin (French part), Channel Islands, Liechtenstein, Sweden, Chile, Lithuania, Switzerland, Croatia, Luxembourg, Taiwan China, Curaçao, Macao SAR China, Trinidad and Tobago Cyprus, Malta, Turks and Caicos Islands, Czech Republic, Monaco, United Arab Emirates, Denmark, Nauru, UK, Estonia, Netherlands, USA, Faroe Islands, New Caledonia Uruguay, Finland, New Zealand, Virgin Islands (USA), France, Northern Mariana Islands French Polynesia, Norway, Germany, Oman, Gibraltar, Palau [68].	Healthcare system in middle and low income countries.
Language	English only	Literature not available in English
Year	No limitations	No limitations
Types of Evidence	Empirical literature and grey literature, of any study design.	Commentaries, guidelines and/or, literature reviews

Articles will be excluded if they present commentaries, guidelines and/or, literature reviews and the research focused solely on undergraduate nurses who have yet to practice. Studies not available in English and in full text will be excluded

The inclusion/exclusion criteria will be piloted on a random set of search results (25% of the results) to ensure strong inter-rater reliability among screeners [70–72]. Once a kappa statistic of >0.80 (almost perfect agreement) [73, 74], is determined between all reviewers, two reviewers will independently screen titles and abstracts of all articles against the inclusion criteria. As suggested by Levac et al. [57], the inclusion and exclusion criteria can be iteratively modified. As such, we will hold regular team meetings to discuss study inclusion/exclusion during the piloting process to determine the final inclusion criteria. The process will be repeated for full-text review, whereby two reviewers will independently review articles deemed eligible at the title and abstract screening. All discrepancies will be resolved through discussion. Where consensus cannot be obtained, a third reviewer will resolve any disagreements relating to study eligibility at both stages (i.e., title, abstract, and full text). We will present our search in a PRISMA flow diagram [59].

### Step 4: Charting the data

The research team will create a data charting form in Covidence to extract data related to the RQs [57], including authors, publication year, type of article, study design, geographical location of the study, description of the nursing practice setting, type of nurse practice activities and characteristics of practice activities, type of support needs, the method to evaluate perceived experiences and needs (i.e., analysis approach), the study findings and limitations of the article.

Two team members (KMK and MS) will test the data extraction form before reviewing all articles independently to ensure sufficient details are captured [57]. The final data extraction form will be shared with the team to ensure all authors’ perspectives of what is important to capture are incorporated. Regular team meetings will be held during extraction to begin the discussion of preliminary thoughts on the data and to resolve any uncertainty about the extracted data.

Although methodological quality is not used as an exclusion criterion in scoping reviews [56], we will assess the quality of included peer-reviewed papers using the Mixed Methods Appraisal Tool (MMAT–Version 2018) [75, 76]. This scoring system was originally designed for systematic reviews that include qualitative, quantitative, and mixed-methods [76]. Two authors trained in mixed-method methodologies and critical appraisals (KMK and MS) will independently complete the quality appraisal and reach a consensus on the quality rating for all included papers in consultation with a third trained author (RA). The purpose of the quality appraisal is to help inform future research directions for high-quality research (RQ3).

### Stage 5: Collating, summarising and reporting the results

We will analyze extracted data using a numerical summary analysis and qualitative thematic analysis guided by Braun and Clarke [77, 78]. First, the research team will independently read the charted data and the included articles. The research team will annotate the articles and identify broad topic categories during the reading process. Each article could have multiple categories based on its contents. The research team will then meet to discuss their thoughts on the data. A codebook will be established [76] through a series of meetings. Next, one author (KMK) will apply the codebook to all articles using NVivo software to manage and organize the coding process [79]. One other author will review the coded data (MS) to establish trustworthiness. Next, the two authors (KMK and MS) will compare and contrast the coded data across the various studies to identify recurrent themes, discrepancies, and unique findings. These findings and themes will be shared with the broader research team over several meetings. During the meetings, we will also consider the implications of our findings on practice, policy and future research [57]. Meetings will occur once a consensus on the final results is identified.

### Stage 6: Consultation

For the consultation step process, we will present our findings to stakeholders invited to participate in a working group meeting to set research priorities for IENs in the context of geriatrics. To ensure a diversity of perspectives, IENs, nurses, educators, and nursing managers from diverse practice settings and cultural backgrounds will be invited to participate in the consultation meeting. We hope to have a representative sample of participants from health care, government, research, academic and policy environments. Ethics approval for the consultative activity will be obtained from [Blinded for Review]. The themes resulting from the review will be presented to participants, who can offer comments on the themes and sub-themes. The expected outcomes of the consultation exercise will be (1) confirmation of the findings and suggestions to enhance the relevance of these findings in future research and program outputs (2) generation of a final set of recommendations for future research. The consultation session will be conducted, recorded and transcribed on Zoom [80]. The comments from the consultative sessions will then be analyzed using an interpretive thematic analysis approach, using a hybrid inductive-deductive thematic analysis technique [81]. The thematic analysis results will be incorporated into the suggested themes presented in this review.

## Limitations

The following limitations constrain this review. First, only literature available in English conducted in high-income countries will be assessed due to resource constraints. Limiting the inclusion criteria to studies published in the English language might pose a limitation by potentially excluding relevant contributions available only in other languages. Although we anticipate that a majority of the relevant literature is in English, this criterion might inadvertently overlook valuable insights or perspectives present in non-English publications. Moreover, we are only reviewing a subset of the grey literature available. Combined, these methodological decisions mean there may be missed sources. Third, our review will be limited to the publicly available literature; thus, additional experiential literature from not publicly available organizational documents will be missed. Lastly, despite including a stakeholder consultative session, stakeholders (e.g., IENs) were not involved in the design of this review protocol.

## Discussion

This study will be the first step to developing a research agenda to improve the experiences and support the needs of IENs in geriatrics. Our aim is to use the findings from this scoping review to guide future research and training by providing a more in-depth understanding of the experiences of IENs caring for older adults. As IENs employed in health settings caring for older adults are often part of interdisciplinary teams, findings from our scoping review will also benefit the broad range of geriatric health care professionals with whom IENs work. The results of this scoping review study will be disseminated through a peer-reviewed publication, a conference presentation, and stakeholder consultations that engage an audience of geriatric practitioners and nursing leaders. As such, findings from the proposed review can inform the development of recommendations, practices and training opportunities for support interventions to help attract and retain IENs.

## Supporting information

S1 File Search strategy. (DOCX)
