# Supplementary material for: Experiences of internationally educated nurses working with older adults: A scoping review protocol
Source: PLoS One. 2024 Oct 3;19(10):e0307795. doi: 10.1371/journal.pone.0307795 (PMC11449320; doi:10.1371/journal.pone.0307795)
Supplement: S1 File — (DOCX) [file pone.0307795.s001.docx]

1 (international* adj2 nurs*).tw,kw,kf. (1979)

2 (internationally educated nurs* or internationally-educated nurs* or internationally trained nurs* or internationally-trained nurs* or internationally educated RN* or internationally-educated RN* or internationally trained RN* or internationally-trained RN* or internationally qualified nurs* or internationally-qualified nurs* or internationally qualified RN* or internationally-qualified RN* or IEN* or IQN*).tw,kw,kf. (1014)

3 Nurses, International/ (406)

4 ((multicultural or foreign* or international* or immigr*) adj2 (nurs* or RN*)).tw,kw,kf. (2723)

5 or/1-4 (3846)
